# *In Situ* Synthesis Mechanism and Photocatalytic Performance of Cyano-Bridged Cu (I)/Cu (II) Ultrathin Nanosheets

**DOI:** 10.3389/fchem.2022.911238

**Published:** 2022-06-20

**Authors:** Shixiong Li, Jiawei Qiang, Lifei Lu, Shaolong Yang, Yufeng Chen, Beiling Liao

**Affiliations:** ^1^ School of Mechanical and Resource Engineering, Wuzhou University, Wuzhou, China; ^2^ School of Chemistry and Chemical Engineering, Guangxi University, Nanning, China; ^3^ School of Chemistry and Biological Engineering, Hechi University, Hechi, China

**Keywords:** photocatalyst, *in situ* synthesis, mechanism, Cu (I)/Cu (II), nanosheets

## Abstract

*In situ* synthesis of cyano-bridged Cu (I)/Cu (II) complexes usually requires organometallic catalysts or is carried out under high-temperature and high-pressure conditions. Herein, the cyano-bridged two-dimensional Cu (I)/Cu (II) photocatalyst, [Cu_2_ (Py)_3_(CN)_3_]_n_ (**1**), is synthesized *in situ* at room temperature. The *in situ* synthesis mechanism of **1** shows that the partial Cu (II) complex catalyzed the C-C bond cleavage of 1,3-isophthalonitrile (L) to introduce -CN and generate Cu (I)/Cu (II). Its ultrathin nanosheets can be obtained by adding sodium dodecyl benzene sulfonate and performing ultrasonic synthesis in the process of synthesis **1**. The ultrathin nanosheets of **1** have a lattice distance of about 0.31 nm, and it can rapidly decompose methylene blue (MB) (K = 0.25 mg L^−1^ min^−1^ at pH = 3). This research work is beneficial for *in situ* synthesis of cyano-bridged Cu (I)/Cu (II) complexes at room temperature and explores their synthesis and photocatalytic mechanism.

## Introduction

The full use of renewable solar energy can solve the problems of environmental pollution and energy shortage (Liu L. et al., 2021; Wei et al., 2021). One of the conditions affecting the photocatalytic degradation of organic pollutants is the efficient use of light (Wang et al., 2021a; Liu X. et al., 2021; Karthik et al., 2022). Tuning the energy-level distribution of photocatalysts at the molecular level can expand their light absorption range and utilization efficiency to improve the photocatalytic performance. The organic ligands that contain unsaturated functional groups can effectively improve the absorption and utilization of light (Tanabe and Cohen, 2011; Mei et al., 2021). Cyano (-CN) is one of the organic ligands that contain unsaturated bonds. Introducing it into photocatalysts could expand the absorption and utilization of light (Liu et al., 2018; Wang et al., 2021b; Pan et al., 2022). However, its introduction into materials often requires the use of toxic cyanides (Li et al., 2018a) [such as KCN, NaCN, Zn (CN)_2_, TMSCN, and K_3_Fe (CN)_6_]. These toxic cyanides pose a serious threat to the environment and even humans if used improperly. *In situ* introduction of -CN is a method that can reduce the use of toxic cyanide. Catalytic C-C bond cleavage of acetonitrile molecules to introduce -CN is a commonly used method (June 2004; Lu et al., 2004; Li et al., 2015; Xia et al., 2016). This reaction usually requires the use of organometallic catalysts (Murahashi et al., 1986; Luo et al., 1998; Taw et al., 2002; Tobisu et al., 2006; Yasui et al., 2008; Grochowski et al., 2010; Xu et al., 2012), for example, [Pd(PPh_3_)_4_], [Cp (PMe_3_)Rh(SiPh_3_)(CH_2_Cl_2_)][BArˊ_4_], and [RhCl(cod)]_2_. Some Cu (Marlin et al., 2001; Zhang and Fang, 2005; Li et al., 2009; Xu et al., 2010; Zhu et al., 2011; Xu et al., 2013), Zn (Yang et al., 2009), and Ag (Huang et al., 2004; Guo et al., 2009) complexes can also perform this reaction at high temperature and high pressure. However, whether using organometallic catalysts or using high temperature and pressure, they consume a lot of energy. *In situ* synthesis of -CN-bridged complexes at room temperature can save energy and discover new synthetic mechanisms.

In mixed valence metal complexes (Heyduk and Nocera, 2001; Yue et al., 2016; Dao and Sun, 2021), since they contain metals in two valence states, electron transfer easily occurs between these two valence states and has important research values such as electronic conductivity and characteristic color changes. Since mixed valence complexes contain ions of the same element with different oxidation states, the charges are transferred between ions of different oxidation states under the action of an external electric field, so mixed valence complexes generally have good electronic conductivity. Moreover, most of the mixed valence complexes have strong absorption in the visible light region and show a darker color, which can be used to develop photocatalytic materials to expand the absorption of light. Research has shown that there are many mixed valence complexes, such as Pt (II, III), Ru (II, III), Os (II, III), Fe (II, III), and Mn (III, IV) complexes. The Cu (I)/Cu (II) complexes are mostly monovalent and divalent, with a 3d^9^/3d^10^ configuration. In Cu (I)/Cu (II) complexes, the copper (I) and copper (II) centers have different coordination numbers and steric structures, which lead to their easy formation of complexes with two-dimensional (2D) structures. With full exposure to surface atoms and/or active sites, 2D materials nanosheets (Wang et al., 2020; Zhao et al., 2020; Thirumal et al., 2021) can be advantageous for improving the catalytic performance.

In this study, a cyano-bridged 2D Cu (I)/Cu (II) photocatalyst, [Cu_2_ (Py)_3_ (CN)_3_]_n_ (**1**), is synthesized *in situ* by C-C bond cleavage of 1,3-isophthalonitrile (L) at room temperature. The *in situ* synthesis mechanism of **1** is analyzed by X-ray single-crystal diffraction, Fourier infrared (IR), electrospray ionization mass spectrometry (ESI-MS), and electron paramagnetic resonance (EPR). Mechanistic inference and active species identification for the photocatalytic degradation of methylene blue by **1** are carried out by using ESI-MS and EPR. The aforementioned results provide a direction for the *in situ* synthesis and explore the mechanism of cyano-bridged Cu (I)/Cu (II) complexes at room temperature.

## Experimental Section

### Materials and Methods

1,3-Isophthalonitrile (L), P25, Cu (NO_3_)_2_·3H_2_O, ZrCl_4_, N,N-Dimethylformamide (DMF), sodium dodecyl benzene sulfonate, 2-aminoterephthalic acid, pyridine (Py), anhydrous methanol, and methylene blue (MB) are analytically pure and purchased from Energy Chemical (Shanghai, China). All the water in the experiment is pure water produced by Wahaha Company (Hangzhou, China).

The IR spectra of **1** are measured on KBr pellets with a Nicolet 5DX FT-IR spectrometer. The elemental analysis of **1** (carbon, hydrogen, and nitrogen) is performed with a Perkin-Elmer 240 elemental analyzer. The X-ray phase analysis of **1** is carried out using Rigaku’s D/max 2500 X-ray diffractometer with Cu Kα radiation (λ = 0.15604 nm); the tube voltage was 40 kV, the tube current was 150 mA, a graphite monochromator was used, and 2θ was 5° to 65°. The X-ray single-crystal diffraction of **1** is obtained on a Bruker Apex CCD area-detector diffractometer. The X-ray photoelectron spectroscopy (XPS) measurements of **1** are performed on a Kratos Axis Ultra DLD system with a base pressure of 10^−9^ torr. Scanning electron microscopy (SEM) of **1** is performed by using a Hitachi S-4800 under the following conditions: Mag.: 1 KX, signal A: VPSE, and EHT: 20 kV. The thermal stability of **1** is tested on a Pyris Diamond TG-DTG Analyzer. Ultraviolet–visible (UV-Vis) absorption spectroscopy of **1** is performed using a UV-2700 instrument from Shimadzu of Japan with BaSO_4_ as a reference. The concentration of MB in the solution was measured with a UV-Vis 2550 at a 664 nm wavelength.

### *In Situ* Synthesis of [Cu_2_ (Py)_3_ (CN)_3_]_n_ (1)

A mixture of Cu (NO_3_)_2_·3H_2_O (0.0723 g, 3 mmol), 10 ml of water, and 10 ml of methanol was used to obtain a light-blue solution. Then 5 ml of pyridine is added to the solution, and the color of the solution changed to navy blue. Finally, 5 mg of 1,3-isophthalonitrile (L) is added to the solution and stirred magnetically for 3 h. The solution is naturally volatilized for 1–2 months, and yellow bulk crystals of **1** are obtained. Yield: 25% (based on copper). Anal. Calcd for C_18_H_15_Cu_2_N_6_: C, 48.82; H, 3.39; N, 18.98. Found: C, 53.47; H, 3.14; N, 18.35. IR (cm^−1^): 3442 s, 2118 s, 1724 w, 1600 m, 1442 s, 1390 w, 1213 w, 1147 w, 1068 w, 767 m, 701 s, 491 w, and 426 w.

### Synthesis of Nanosheets of 1

The front part of the synthetic nanosheets is the same as the synthesis of [Cu_2_ (Py)_3_ (CN)_3_]_n_ (1). The filtrate was collected in 50 ml of a beaker, 2 ml of 50 mg/L sodium dodecyl benzene sulfonate was added, and then ultrasound was applied for 10 min; The suspension was collected and centrifuged at 10,000 rpm. The solvent molecules were removed and washed with anhydrous methanol and water three times and dried in an oven at 80°C for 8 h. The nanosheets of **1** were obtained (yield: 91%, based on copper).

### Synthesis of UIO-66-NH_2_

A mixture of ZrCl_4_ (3.495 g, 15 mmol) and 2-amino terephthalic acid (2.715 g, 15 mmol) was dissolved in 115 ml of DMF with the aid of ultrasonic vibrations. The reaction mixture was heated at 120°C for 24 h and then cooled to room temperature. The solvent was removed, and the solid powder was washed three times with DMF and methanol and dried at 80°C for 8 h to obtain UIO-66 as a powder. Yield: 88% (based on p-phthalic acid).

### X-Ray Crystallography

X-ray crystallography is performed on a Bruker Apex CCD area-detector diffractometer (Mo*K*_α_, λ = 0.71073 Ǻ), and the structure was solved by direct methods using the Olex^2^ program and refined with the Olex^2^ program (Dolomanov et al., 2009). The hydrogen atoms were placed at calculated positions and refined as riding atoms with isotropic displacement parameters. Crystallographic crystal data and structure processing parameters for **1** are summarized in Supplementary Table S1. Selected bond lengths and bond angles for **1** are listed in Supplementary Table S2. Supplementary Material for **1** has been deposited with the Cambridge Crystallographic Data Centre [CCDC nos. 2141770 (**1**); deposit@ccdc.cam.ac.uk or http://www.ccdc.cam.ac.uk].

### Preparation of Solutions for Electrospray Ionization Mass Spectrometry

The MB solution after photocatalytic degradation for 60 min is centrifuged to remove **1**, and the collected solution is subjected to rotary evaporation to remove water molecules. Then 2 ml of anhydrous methanol was added to prepare a solution that could be used to analyze the photocatalytic degradation of MB products.

### Photocatalytic Experiments

Taking photocatalytic degradation of MB as an example, the photocatalytic performances of **1** and its nanosheets are investigated and compared with those of P25 and UIO-66-NH_2_. The photocatalytic reaction conditions are as follows: the MB initial concentration C_0_ = 5 mg/L, pH 3–9, dosage 50 ml; photo-irradiation is carried out using a 300 W xenon lamp through UV cut-off filters to completely remove any radiation below 420 nm and to ensure illumination by visible light only. The irradiation intensity is approximately 10 W/m^2^; a 250 ml round glass container with a circulating water jacket is used, temperature (T) = 25°C; the weights of the **1**, nanosheets, P25, and UIO-66-NH_2_ are 50 mg. The concentration of MB was measured using a UV-Vis 2550 at a 664 nm wavelength. The photocatalytic degradation performance is evaluated through the changes in the concentration of MB before and after the reaction. The degradation rate was calculated as follows:

$$\text{η}=\frac{{\text{C}}_{0}-\text{C}}{{\text{C}}_{0}}\times 100\%$$

where η is the degradation rate (%) and C_0_ and C are the qualities of performance before and after degradation, respectively (mg/L).

## Results and Discussion

### Structure of 1

**FIGURE 1:**
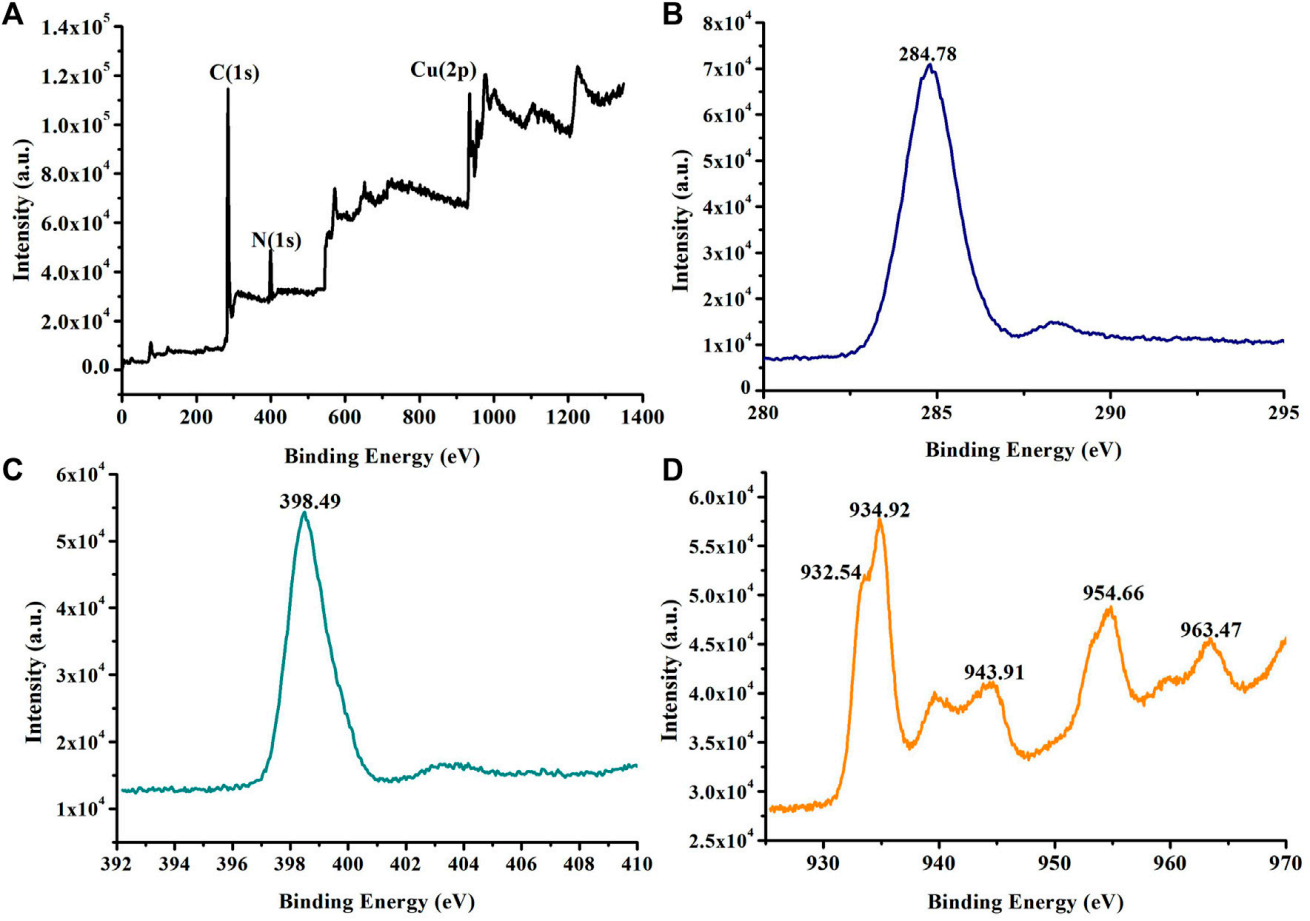
XPS of **1**: **(A)** survey, **(B)** C (1 s), **(C)** N (1 s), and **(D)** Cu (2p).

In the previous work (Li et al., 2019a; Li et al., 2019b; Li et al., 2021), we found that Cu (I) and Cu (I)/Cu (II) complexes can be synthesized *in situ* by controlling the molar ratio of reactants, reaction temperature, and time under high-temperature and high-pressure hydrothermal synthesis conditions. Herein, at room temperature, Cu (NO_3_)_2_·3H_2_O, pyridine (Py), and 1,3-phenylenediacetonitrile (L) are reacted in a mixed solution of water and methanol to obtain a yellow block coordination polymer [Cu_2_ (Py)_3_ (CN)_3_]_n_ (**1**). EPR (Supplementary Figure S1) indicated that **1** contained a single electron, which is possibly from Cu (II) or Cu (IV). To further analyze the valence state of Cu in **1**, XPS characterization is performed. XPS (Figure 1A) and energy-dispersive X-ray spectroscopy (EDS) mapping (Supplementary Figure S2) show that **1** is composed of the C, N, and Cu elements. The XPS characteristic diffraction peaks of C (1 s) (Figure 1B) and N (1 s) (Figure 1C) are found at 284.78 (eV) and 398.49 (eV), respectively. They are in the range of theoretical and literature reported diffraction peaks (Li et al., 2018b). However, for the valence state of copper in **1**, this did not provide valuable information about Cu (IV) ions but further determined the possibility of magnetic contamination having a 3d^9^ configuration. Both Cu (I) and Cu (II) signals were observed (Figure 1D, Supplementary Figure S3): the Cu (II) has a main peak at 934.92 eV (peak II) with a shakeup satellite 943.5 (eV) (peak III) at higher binding energies, and Cu(I) has a characteristic peak at 932.54 eV (peak I) with no satellite peak (Li et al., 2018c).

The Fourier IR (Supplementary Figure S4) of **1** shows that it has a strong absorption at 2118 cm^−1^, which can be assigned to the characteristic absorption peak of -CN (Marlin et al., 2001; Zhang and Fang, 2005; Li et al., 2009; Xu et al., 2010; Zhu et al., 2011; Xu et al., 2013). In view of the aforementioned analysis, it can be determined that -CN and Cu (I)/Cu (II) exist in **1**.

**FIGURE 2:**
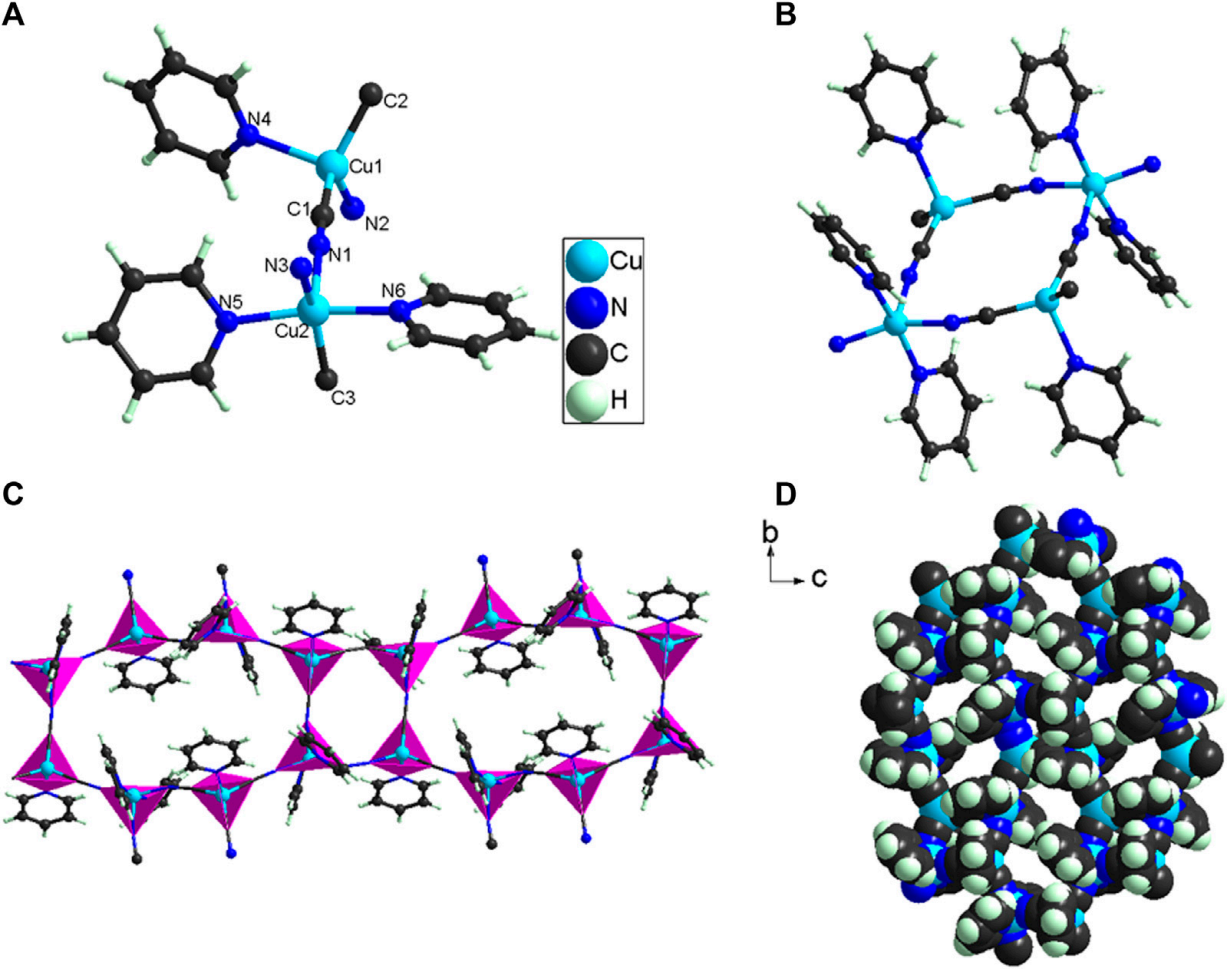
**(A)** The smallest structural unit of **1**; **(B–C)** The 2D structure formed by -CN bridged Cu(I)/Cu( II); **(D)** Hole distribution in 2D structure of **1**.

An X-ray single-crystal diffraction analysis reveals that **1** crystallizes in the monoclinic system, *P2*_*1*_ */c* space group (Supplementary Table S1), with a = 14.5080(7) Å; b = 18.2789(6) Å; c = 14.6503(7) Å; β = 107.953(5)º; V = 3695.9(3) Å^3^. **1** is a coordination polymer (Figure 2), and its smallest structural unit (Figure 2A) shows its molecular formula is C_18_H_15_Cu_2_N_6_, which is mainly composed of one Cu (II) cation, one Cu (I) cation, three pyridine molecules, and three CN^−^ anions. The EPR (Supplementary Figure S1) test result has shown that the coordination environment of Cu (II) ions in **1** does not have axisymmetric properties. Therefore, in **1**, Cu (I) is coordinated with one pyridine molecule and three CN^−^ anions, which belongs to 4-coordination. However, the Cu (II) in **1** is coordinated with two pyridine molecules and three CN^−^ anions, which belongs to 5-coordination. The CN^−^ belongs to bridging ligands, so the CN^−^ in **1** bridges adjacent to Cu (I) and Cu (II) to form a 2D structure (Figures 2B–D). In **1**, it is very interesting that there are two kinds of pores in it (Supplementary Figure S5). One of them is a small hole; it is composed of four -CN-bridged four Cu (I) and Cu (II). The other is a micropore; it is composed of eight -CN-bridged eight Cu (I) and Cu (II). The direct distribution of micropores in **1** is 7.5 × 10.5 Å (Supplementary Figure S5). Although Cu (I) and Cu (II) in **1** are coordinated with multiple CN-, the C-N distances fall in the range of 1.140(5) ∼ 1.159(5) Å [C(1)-N(1) = 1.140(5) Å; C(2)-N(2) = 1.158(5) Å; C(3)-N(3) = 1.159(5) Å]. The Cu-N and Cu-C distances fall in the range of 1.969(3) ∼ 2.158(3) Å and 1.947(4) ∼ 1.969(4) Å, respectively. These bond distances (Supplementary Table S1) and bond angles (Supplementary Table S2) in **1** are comparable to those in other cyanide-bridged copper (I)/Cu (II) complexes (Marlin et al., 2001; Zhang and Fang, 2005; Li et al., 2009; Xu et al., 2010; Zhu et al., 2011; Xu et al., 2013).

### *In Situ* Synthesis Mechanism of 1

The aforementioned EPR, XPS, IR, and X-ray single-crystal diffraction have indicated the elemental composition, functional groups, and valence states of Cu ions in **1**. The solution during *in situ* synthesis of **1** is collected and tested for electrospray mass spectrometry (ESI-MS). Numerous molecular fragmentation peaks (Supplementary Figure S6) appeared in ESI-MS of the solution during *in situ* synthesis of **1**. There are seven molecular fragment peaks related to Cu ions that can be deduced according to the composition and valence state of reactant raw materials and solvents. They are [Cu·(L)·(Py)·(H_2_O)_2_·(NO_3_)]^+^,[Cu_2_·(Py)_3_·(CN)_3_]^+^, [Cu·(L)_2_·(NO_3_)·(H_2_O)_2_]^+^,[Cu·(Py)_4_·(H_2_O)_2_]^2+^,[Cu·(NO_3_)_2_·(H_2_O)_4_]^+^, [Cu·(Py)_2_·(H_2_O)_2_·(NO_3_)_2_]^+^, and [Cu·(L)_2_·(NO_3_)_2_·(H_2_O)_2_]^+^.

**FIGURE 3:**
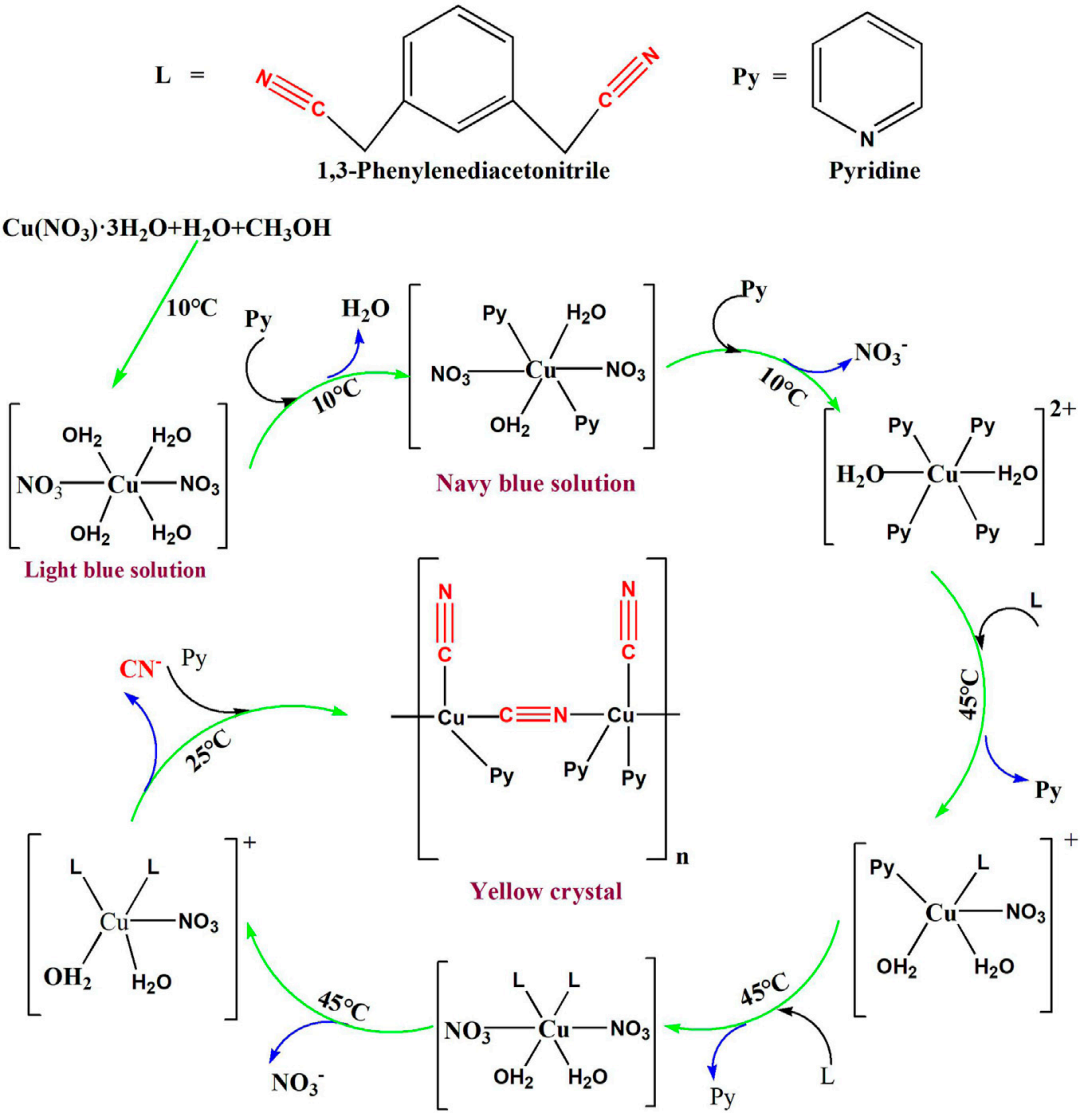
*In situ* synthesis mechanism of **1**.

Therefore, the possible *in situ* synthesis mechanism (Figure 3) of **1** is as follows: at room temperature, Cu (NO_3_)_2_·3H_2_O is dissolved in 1:1 methanol and water to form a light-blue solution, and the temperature of the solution is about 10°C. At this time, the Cu (II) exists in the form of [Cu·(NO_3_)_2_·(H_2_O)_4_] in solution. Then 5 ml of pyridine is added to the solution, and the color of the solution changed from light blue to navy blue. This indicates that pyridine reacts with Cu (II) to form [Cu·(Py)_2_·(H_2_O)_2_·(NO_3_)_2_]. Since the amount of pyridine added to the solution is very large, it has a stronger coordination ability with Cu (II) than NO_3_^−^, thus forming [Cu·(Py)_4_·(H_2_O)_2_]^2+^. Finally, 5 mg of 1,3-isophthalonitrile (L) is added to the navy solution, and the temperature of the solution at this time changed from 10 to 45°C. This phenomenon indicates that the Cu (II) complex has reacted with L and formed [Cu·(L)·(Py)·(H_2_O)_2_·(NO_3_)]^+^ and [Cu·(L)_2_·(NO_3_)_2_·(H_2_O)_2_]. The coordinating atoms in the six-coordinated [Cu·(L)_2_·(NO_3_)_2_·(H_2_O)_2_] are too crowded, so one NO_3_^−^ anion is lost to form [Cu·(L)_2_·(NO_3_)·(H_2_O)_2_]^+^. In [Cu·(L)_2_·(NO_3_)·(H_2_O)_2_]^+^, Cu (II) catalyzes the cleavage of the C-C bond in L, generating (CN)_2_ and 1,3-dimethylbenzene. At this time, the Cu (II) and (CN)_2_ undergo a redox reaction to form Cu (I) and CN^−^, respectively. Therefore, at this time, in addition to a large amount of pyridine, there are Cu (I) and CN^−^ in the solution. They self-assemble at room temperature to form [Cu_2_·(Py)_3_·(CN)_3_]_n_. It can be seen that the copper complex can also catalyze the cleavage of the C-C bond on the L structure and *in situ* synthesis of cyano-bridged Cu (I)/Cu (II) coordination polymers at room temperature.

### Photoelectric Response and Photocatalytic Performance of 1

**FIGURE 4:**
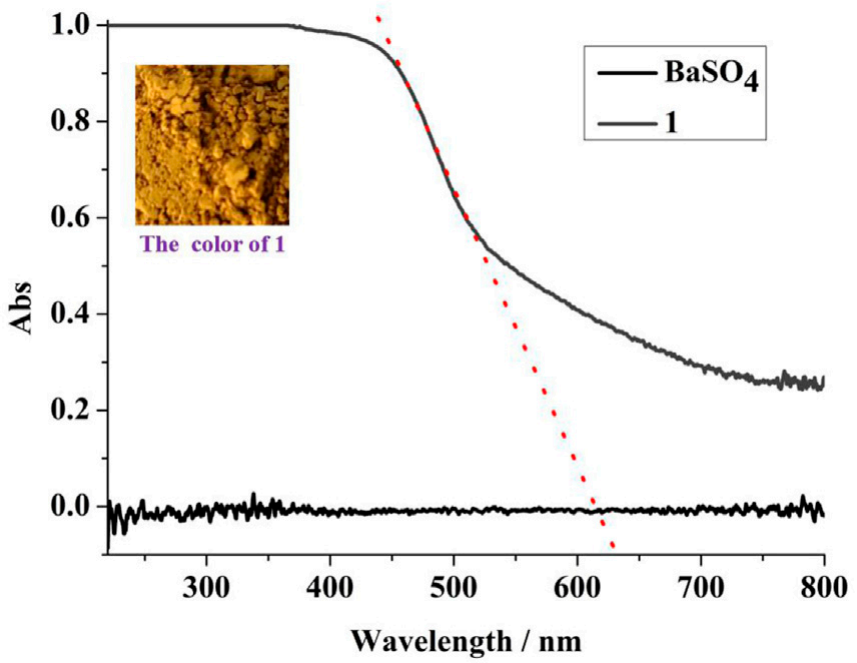
The UV-Vis absorption spectroscopy of **1**.

UV-Vis diffuse reflectance spectroscopy (Figure 4) shows that **1** is yellow, and it has more than 30% light absorption in the range of 220–800 nm. The cyclic voltammetry curve (Supplementary Figure S7) shows that the band gap (eV) of **1** is 2.63 eV. The HOMO = **−**5.41 eV and LUMO = **−**2.78 eV of **1** can be calculated by the following formulas (Li et al., 2018a): E_HOMO_/eV = **−**4.44—Eonset (Ox); E_LUMO_/eV = **−**4.44—Eonset (Red).

**FIGURE 5:**
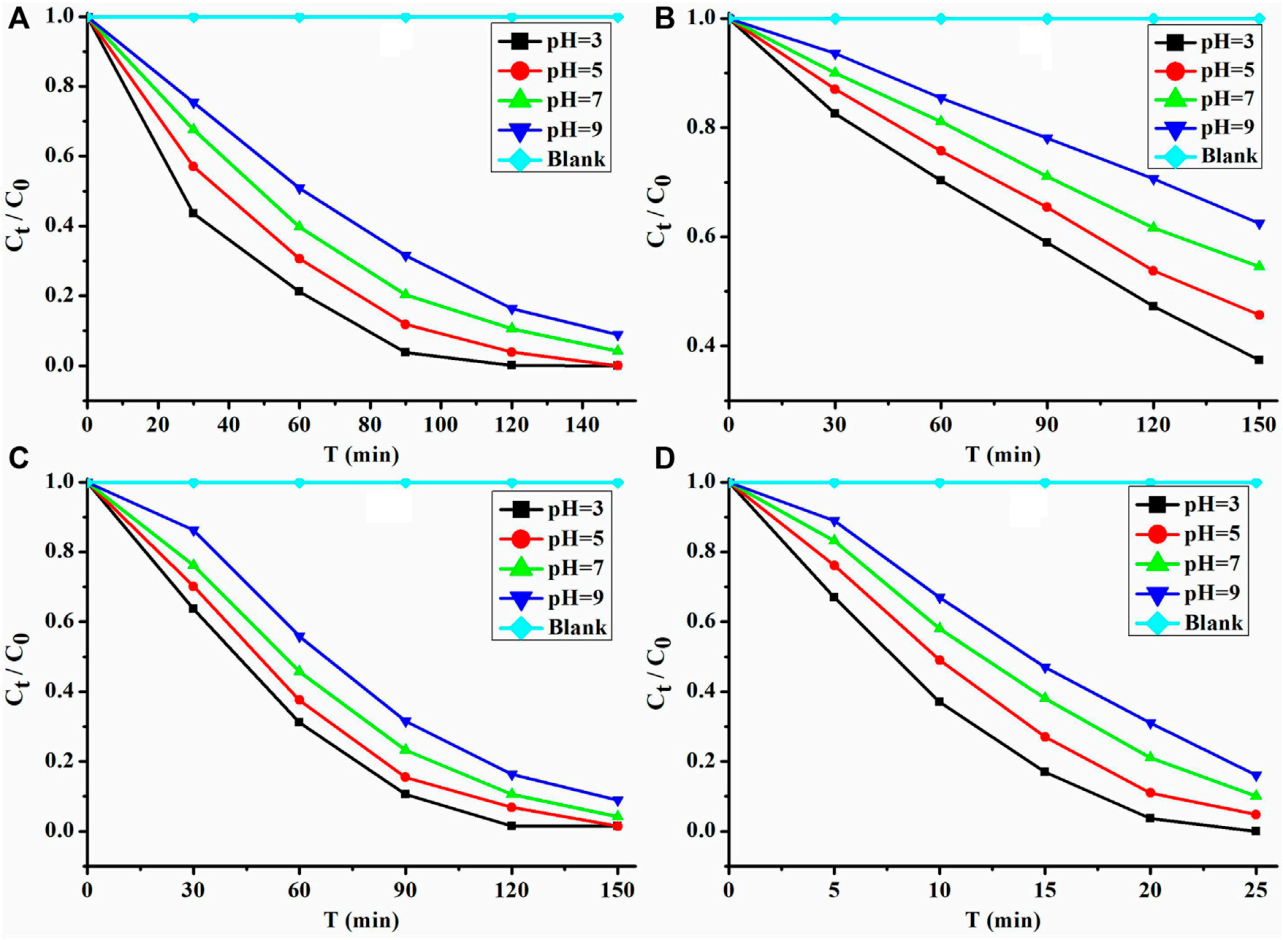
Performance of photocatalytic degradation of MB: **(A) 1**, **(B)** P25, **(C)** UIO-66-NH_2_, and **(D)** nanosheets of **1**.

Therefore, **1** and its nanosheets are very good photocatalysts with a visible light response; they photocatalytically degrade MB in comparison with P25 and UIO-66-NH_2_ in solutions of pH 3–9. The performance of these photocatalysts was investigated under the irradiation of a 300 W xenon lamp, with the MB solution as a blank control. In order to better study the performance of these photocatalysts in photocatalytic degradation of MB, a control experiment of the adsorption of MB is carried out in the dark. The experimental results (Figure 5) show that the amount of photocatalytic degradation of MB per unit time of these photocatalysts is greater than that of the adsorption of MB (Supplementary Figure S8). Moreover, their performance for photocatalytic degradation of MB in acidic solutions is better than that in neutral and basic solutions (Figure 5). Among them, their performance is the best at pH = 3. At this time, the photocatalytic degradation rates of MB can reach 0.0556, 0.0261, and 0.0417 mg L^−1^ min^−1^. The performance of **1** is about 2.1 times that of P25 and 1.3 times that of UIO-66-NH_2_.

**FIGURE 6:**
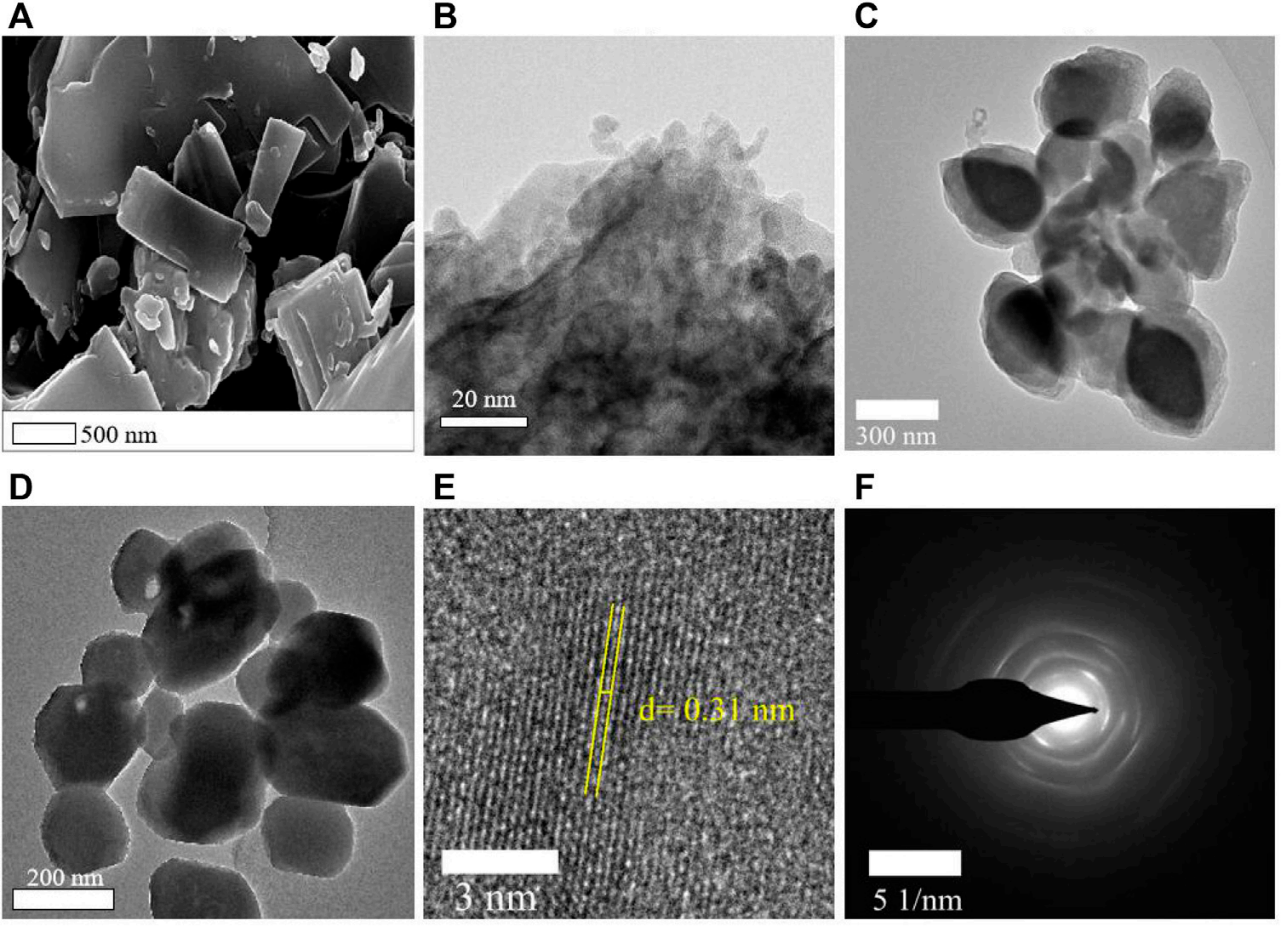
**(A)** SEM of **1** and **(B–F)** TEM of nanosheets.

The ultrathin nanosheets of **1** can be obtained by adding sodium dodecyl benzene sulfonate and performing ultrasonic synthesis in the process of synthesis of **1**. **1** has a 2D structure and is flaky (Figure 6A). When it was exfoliated into nanosheets, its morphology did not change, but it became thinner and has a lattice distance of about 0.31 nm (Figure 6B–F). The EDS mapping (Supplementary Figure S9) of nanosheets shows that it is still composed of the Cu, C, and N elements. Since **1** has become an ultrathin nanosheet, more surface sites can be fully exposed. Therefore, the nanosheets of **1** can quickly decompose MB, and the photocatalytic degradation rate can reach 0.25 mg L^−1^ min^−1^ at pH = 3. This rate is 4.5, 9.6, and 6.0 times that of **1**, P25, and UIO-66-NH_2_, respectively. In addition, the performance of these nanosheets is almost the same as that of MoS_2_ nanosheets (K = 0.50 mg L^−1^ min^−1^ at pH 3). However, the ultrathin Cu (I)/Cu (II) inorganic coordination polymer quantum sheet (ICPQS) {[Cu^II^ (H_2_O)_4_][Cu^I^_4_ (CN)_6_]}_n_ photocatalytic degradation performance (K = 2.5 mg L^−1^ min^−1^ at pH 3) of MB with practical applications (Li et al., 2018b) is 10.0 times that of these nanosheets. However, the performance of these nanosheets for photocatalytic degradation of MB (K = 0.0737 mg L^−1^ min^−1^ at pH 3) is 3.4 times higher than that of the Cu (I) polymer [Cu (L)_2_·(CN)]_n_ (Li et al., 2018c). It can be seen that the exfoliation of 2D materials into ultrathin nanosheets can allow more sites on the catalyst surface to be used, thereby improving the performance of photocatalytic degradation of pollutants.

### Photocatalytic Mechanism of 1

**FIGURE 7:**
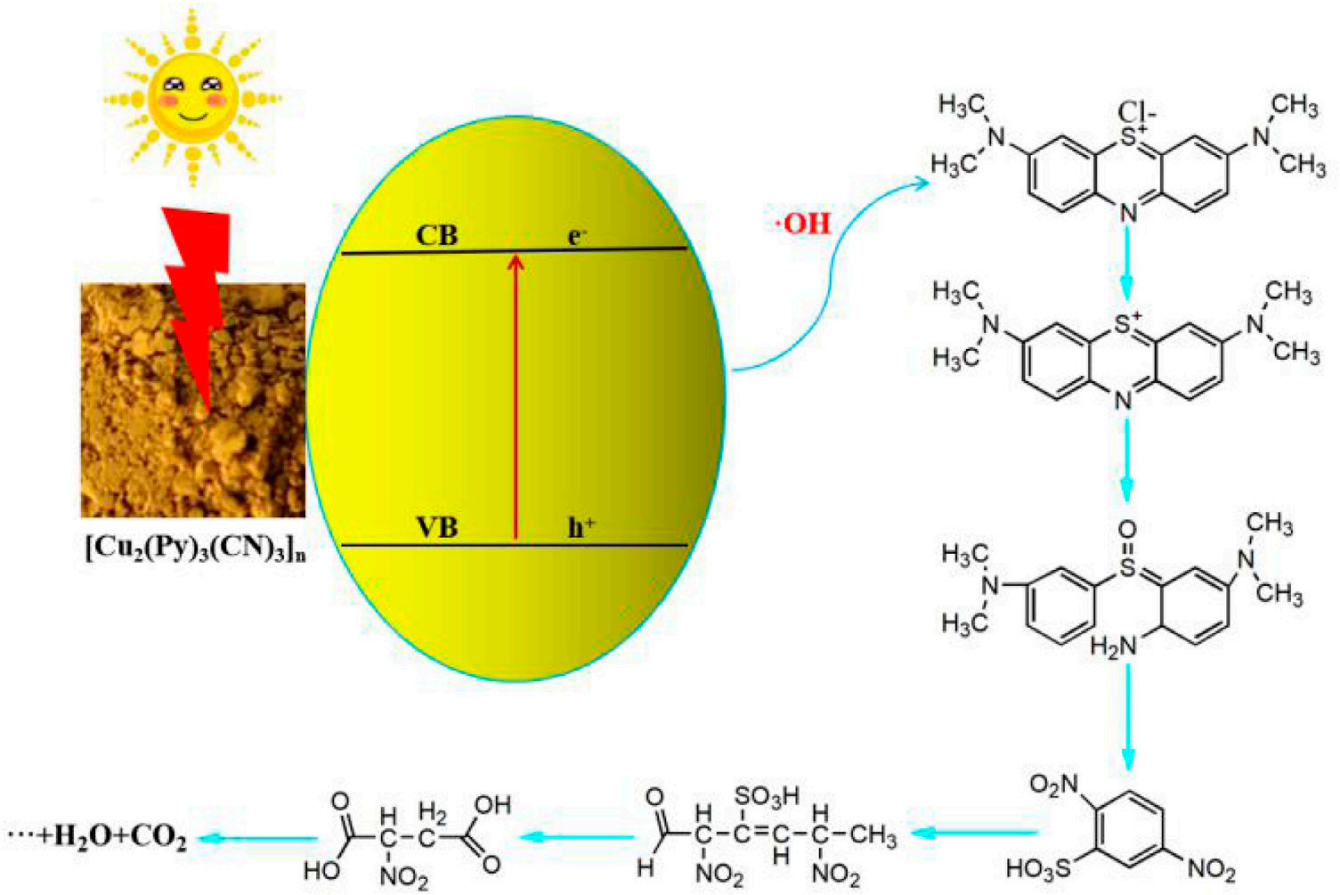
Mechanism of photocatalytic degradation of MB by **1**.

*tert*-Butanol is used as a blank reference. The photocatalytic degradation of MB is carried out by adding dimethyl pyridine N-oxide (DMPO) to the solution at pH = 7, and the free radicals generated during the reaction are detected by EPR. The test results show (Supplementary Figure S10) that the OH active species are generated during the photocatalytic process. The solution of photocatalytic degradation of MB for 60 min is collected for ESI-MS characterization, and the test results show (Supplementary Figure S11) that there are five signals related to the fragmentation of MB molecules. They are [C_16_H_18_N_3_S]^+^, [C_16_H_22_N_3_SO]^+^, [C_6_H_4_N_2_SO_7_]^+^, [C_6_H_9_N_2_SO_8_]^+^, and [C_4_H_6_NO_6_]^+^. Combined with the EPR (Supplementary Figure S10) and ESI-MS (Supplementary Figure S11), the mechanism and path (Figure 7) of **1** photocatalytic degradation of MB can be clearly deduced. It can be clearly seen that under visible light irradiation, the generated OH undergoes a redox reaction with MB, which is carried out in multiple steps: first, the amide bond in the MB molecule is broken; then the S atom and the O atom combine to form a S=O bond; under the attack of free radicals, the methyl group on the N atom is oxidized to generate a small molecular acid and ring opening to generate 2,5-dinitrobenzene sulfonic acid; finally, all aromatic rings are broken off to generate the small molecular acid, CO_2_, and water.

### Stability and Cycling Experiments of 1

**FIGURE 8:**
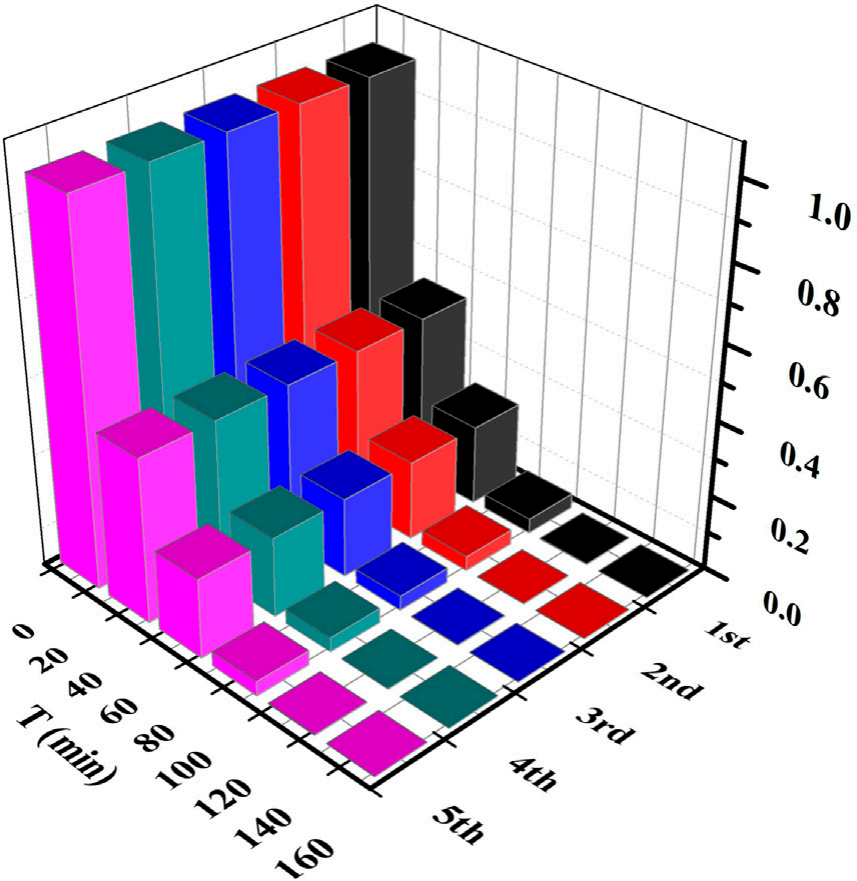
Cycling experiment of **1**.

Both **1** and its nanosheets exhibited good photocatalytic performance under acidic conditions. The photocatalytic degradation of MB is the best at pH = 3 (Figure 5), and their rates can reach 0.0556 and 0.2500 mg L min^−1^, respectively. **1** after photocatalytic degradation of MB is collected and washed with absolute ethanol and water three times. Then it was heated in a blast drying oven at 100°C for 8 h (Supplementary Figure S12 shows that it has good thermal stability within 30–100°C) and characterized by analysis of its powder X-ray diffraction (PXRD). The PXRD shows (Supplementary Figure S13) that its structure has not changed. Therefore, **1** after photocatalytic degradation of MB at pH = 3 is subjected to cycling experiments, and even after five cycles, its performance can still remain above 99.5% (Figure 8).

## Conclusion

In summary, a cyano-bridged 2D Cu (I)/Cu (II) photocatalyst, [Cu_2_ (Py)_3_ (CN)_3_]_n_ (**1**), is synthesized *in situ* at room temperature. Its *in situ* synthesis mechanism suggests that the Cu (II) complex can catalyze the C-C bond cleavage of 1,3-isophthalonitrile (L) to generate -CN and Cu (I)/Cu (II). The photocatalytic degradation of MB by these nanosheets is a multi-step redox process from macromolecules to small molecules with the participation of OH. The results of this study are beneficial to reducing the use of toxic cyanide and *in situ* synthesis of CN^−^ materials at room temperature.

## Data Availability

The original contributions presented in the study are included in the article/Supplementary Material; further inquiries can be directed to the corresponding authors.
