# Supplementary material for: In Situ Synthesis Mechanism and Photocatalytic Performance of Cyano-Bridged Cu (I)/Cu (II) Ultrathin Nanosheets
Source: Front Chem. 2022 Jun 20;10:911238. doi: 10.3389/fchem.2022.911238 (PMC9251208; doi:10.3389/fchem.2022.911238)
Supplement: Supplementary file 1 [file DataSheet1.PDF]

Supporting Materials for

In-situ synthesis mechanism and photocatalytic performance of cyano-bridged Cu(I)/Cu(II) ultrathin nanosheets

Shixiong Li[a,b,*], Jiawei Qiang[a,b] , Lifei Lu[a], Shaolong Yang[a,b], Yufeng Chen[a,] Beiling Liao[c,*]

[a]School of Mechanical and Resource Engineering, Wuzhou University, Wuzhou 543002, P. R. China

[b]School of Chemistry and Chemical Engineering, Guangxi University, Nanning, 530004, P. R. China

[c]School of Chemistry and Biological Engineering, Hechi University, Hechi,Guangxi 546300, P. R. China

*E-mail address: lsx1324@163.com (S. X. Li); liaobeiling1324@163.com (B. L. Liao)

10 pages, 13 figures, 2 tables

CONTENTS

Figure S1. The EPR of **1**. ········ S1

Figure S2. The energy-dispersive X-ray spectroscopy (EDS) mapping of **1**.········ S1

Figure S3.The XPS characterization analysis the Cu valence state in **1**········ S2

Figure S4. The IR of **1**. ········ S2

Figure S5. The -CN bridges adjacent Cu(I) and Cu(II) to form a 2D structure.········ S3

Figure S6. ESI-MS of the solution during *in-situ* synthesis of **1**.········ S3

Figure S7. The CV curve of **1**.········ S4

Figure S8. Performance of adsorption of MB: (a) **1**; (b) P25; (c) UIO-66-NH_2; (d) Nanosheets of **1**.········ S4

Figure S9. EDS mapping of nanosheets. ········ S5

Figure S10. The ·OH reactive species is analyzed by EPR (DMPO trap).········ S5

Figure S11. The ESI-MS of photocatalytic degradation of MB for 60 min by **1**.········ S6

Figure S12. TGA of **1**.········ S6

Figure S13. XRD of **1** collected after the experiment. ········ S7

Table S1. Crystallographic Data of **1**. ········ S8

Table S2. Selected Bond Lengths (Å) and Bond Angles (°) for **1**.········ S9

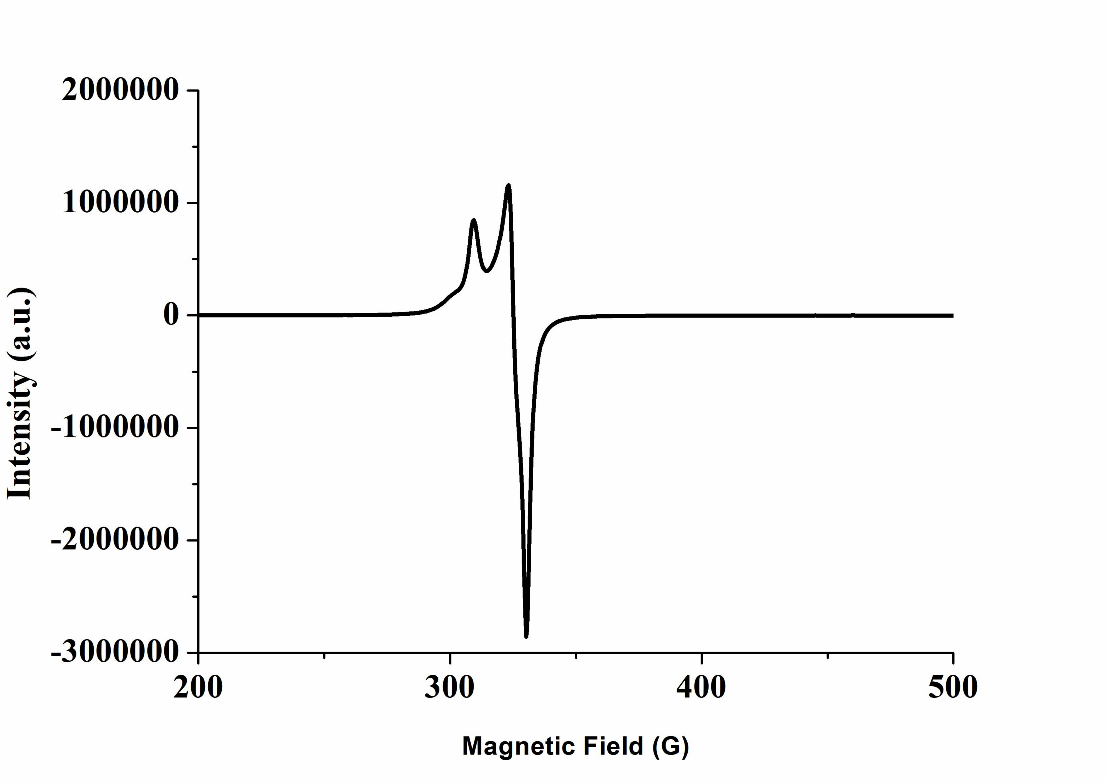

Figure S1. The EPR of **1**.

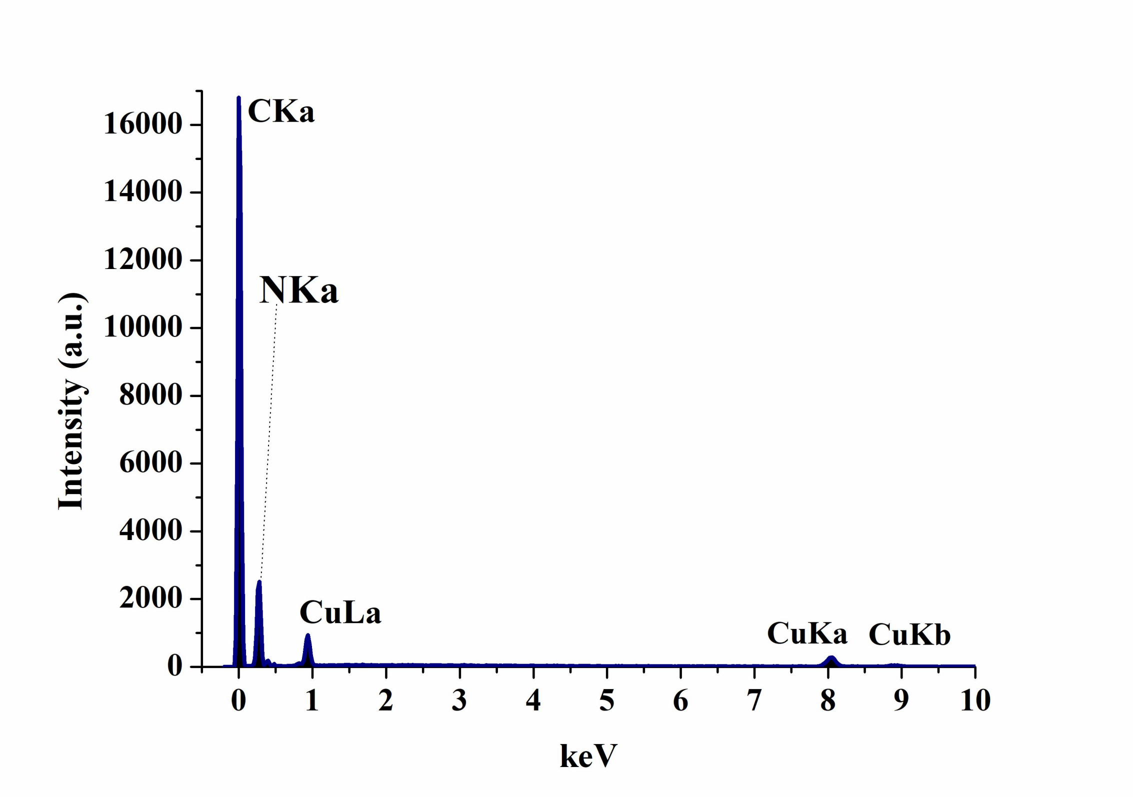

Figure S2. The energy-dispersive X-ray spectroscopy (EDS) mapping of **1**.

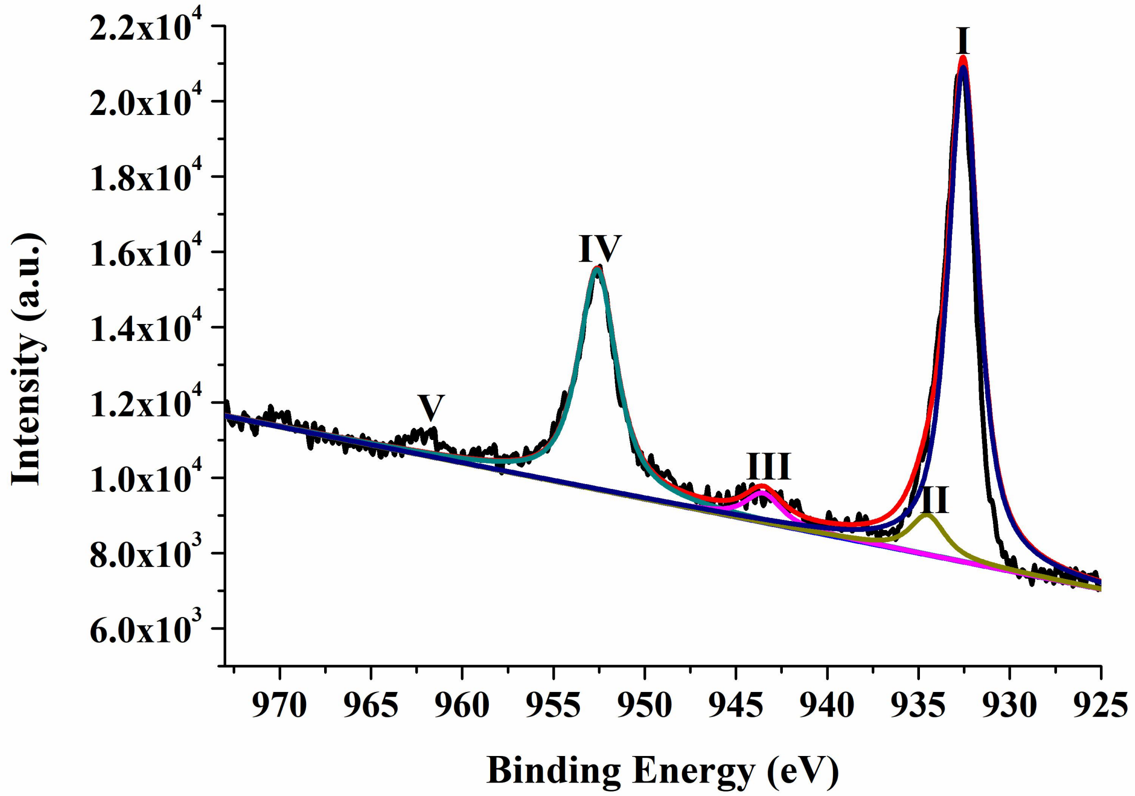

Figure S3. The XPS characterization analysis the Cu valence state in **1**.

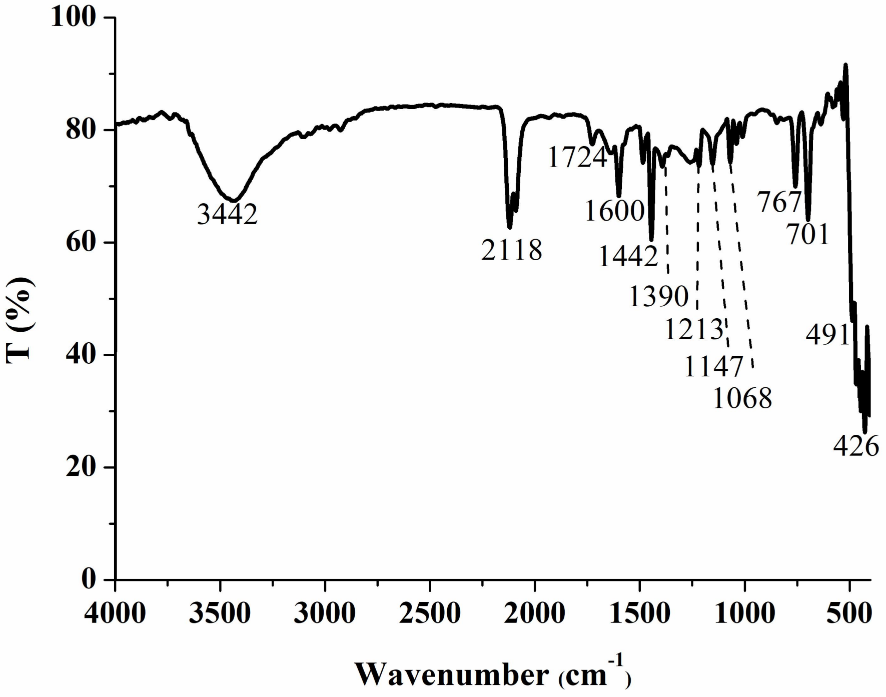

Figure S4. The IR of **1**.

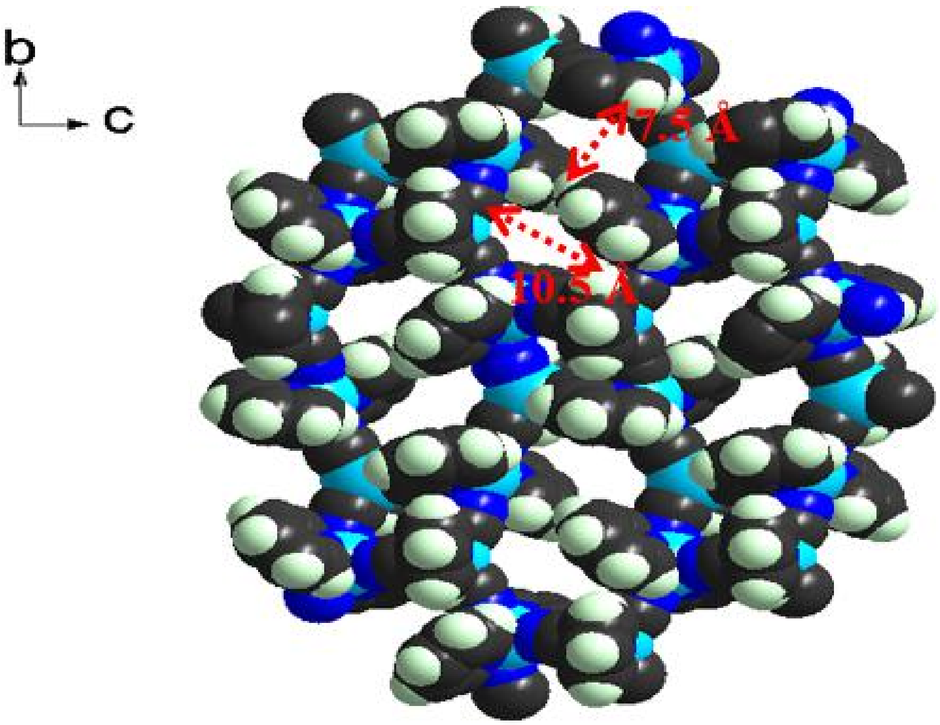

Figure S5. The -CN bridges adjacent Cu(I) and Cu(II) to form a 2D structure.

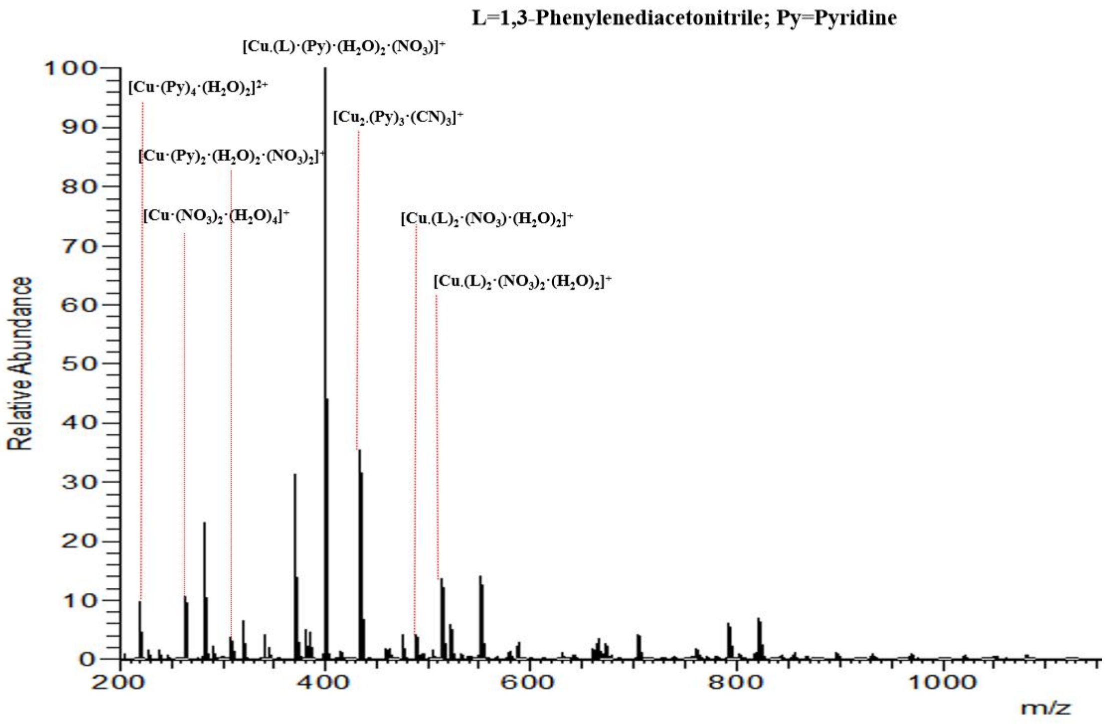

Figure S6. ESI-MS of the solution during *in-situ* synthesis of **1**.

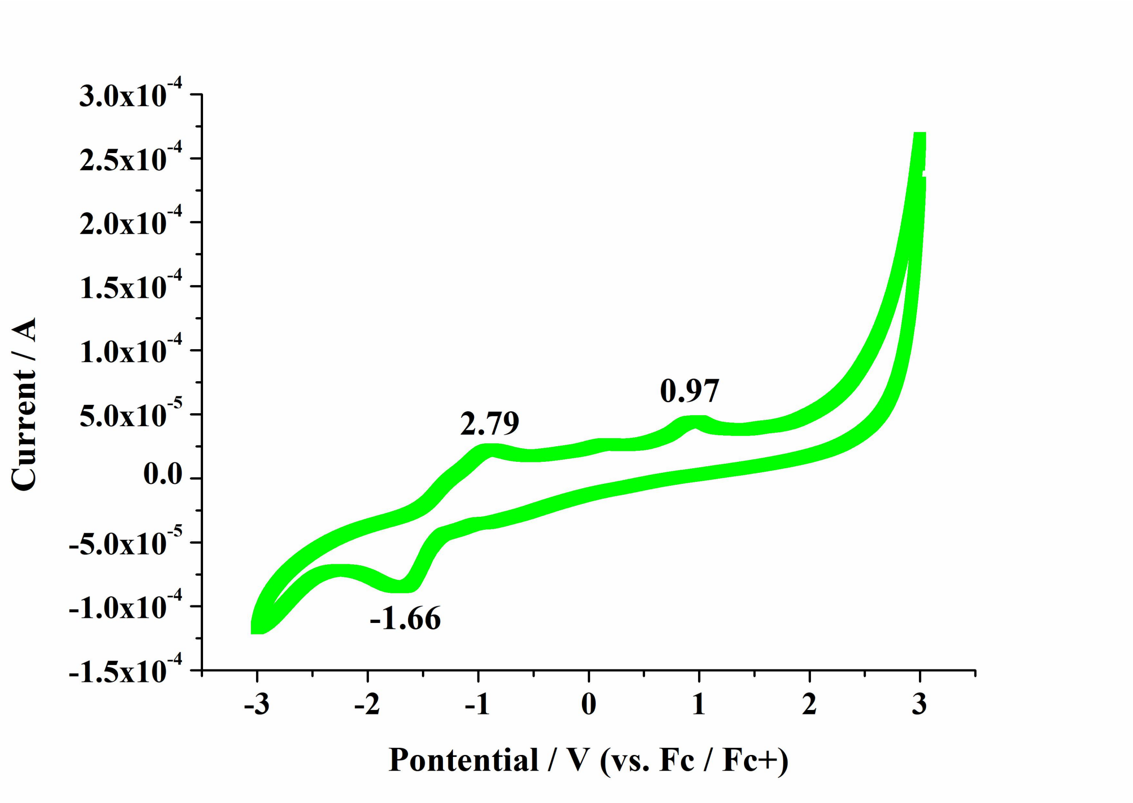

Figure S7. The CV curve of **1**.

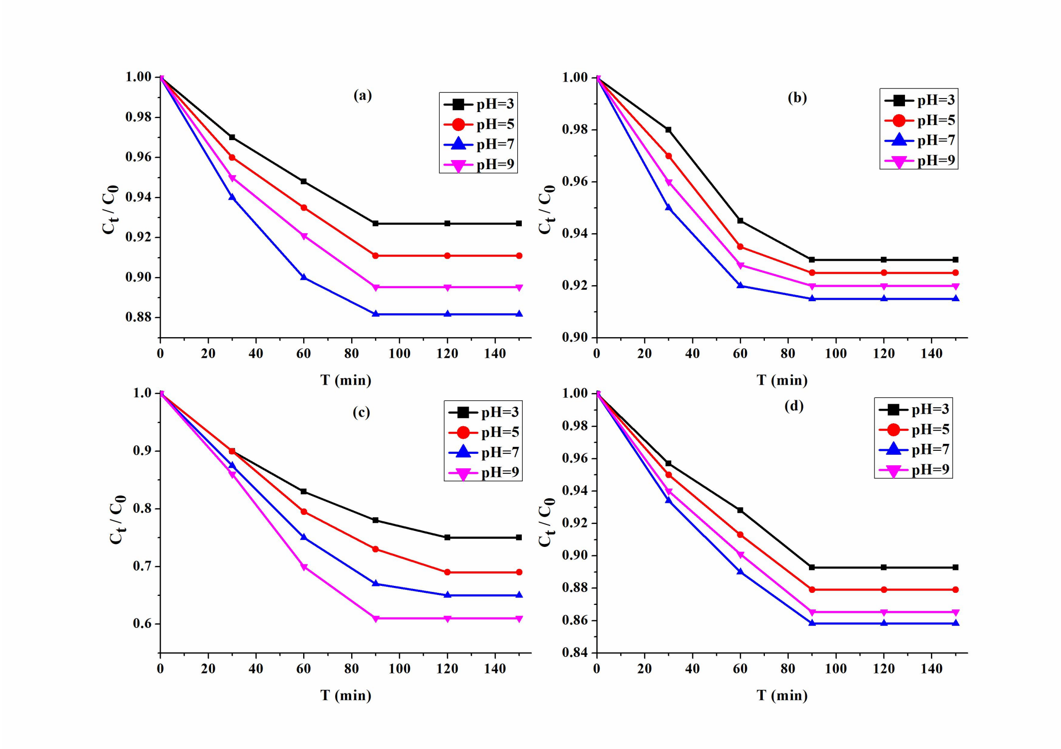

Figure S8. Performance of adsorption of MB: (a) **1**; (b) P25; (c) UIO-66-NH_2; (d) Nanosheets of **1**.

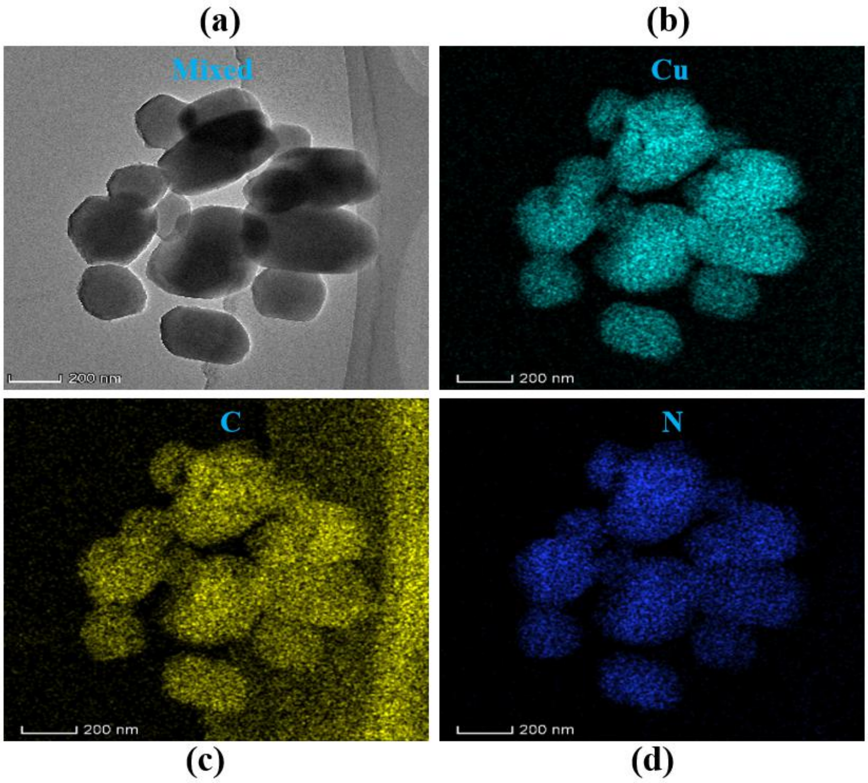

Figure S9. EDS mapping of nanosheets.

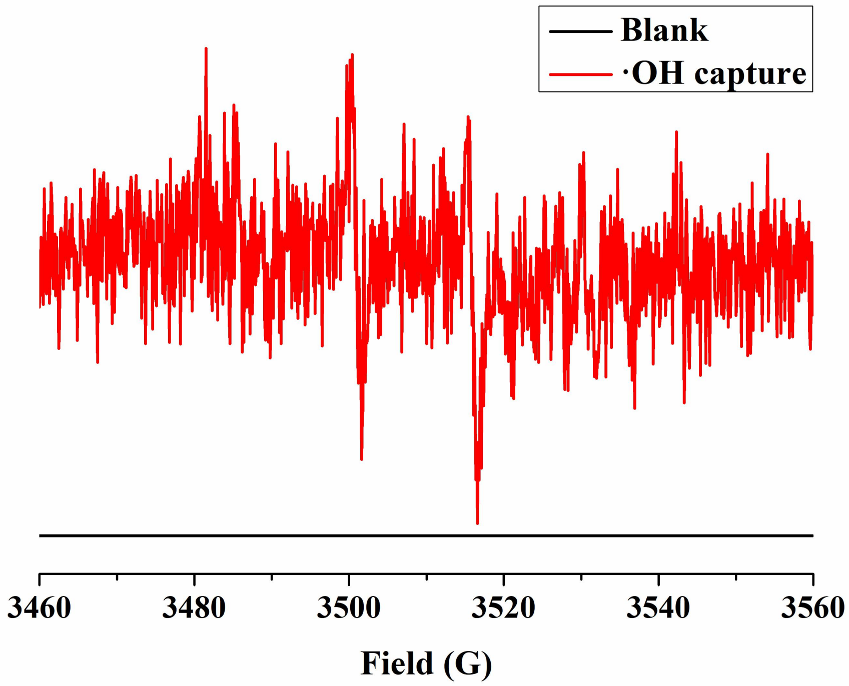

Figure S10. The ·OH reactive species is analyzed by EPR (DMPO trap).

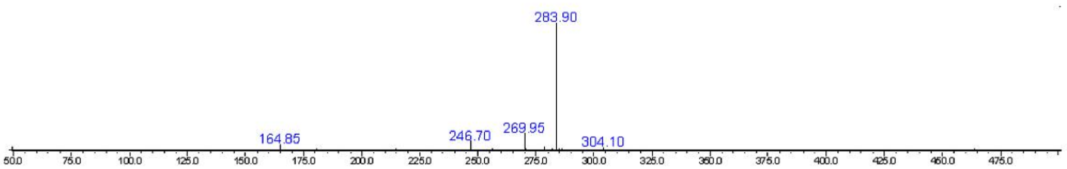

Figure S11. The ESI-MS of photocatalytic degradation of MB for 60 min by **1**.

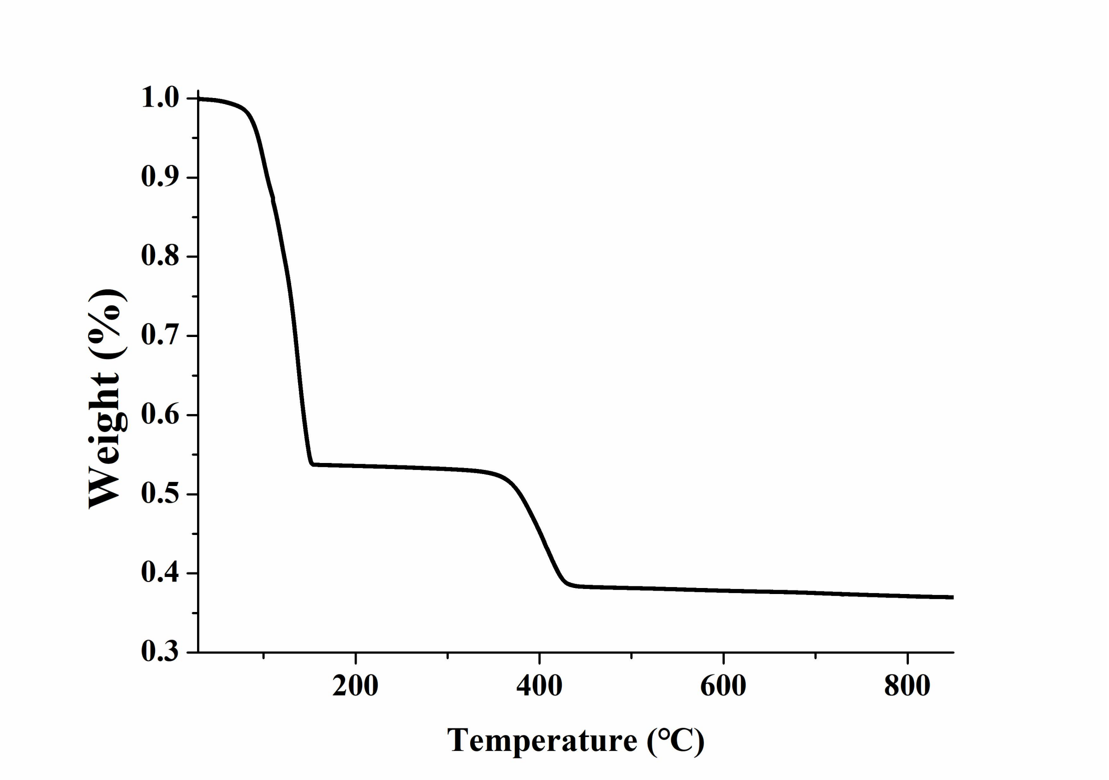

Figure S12. TGA of **1**.

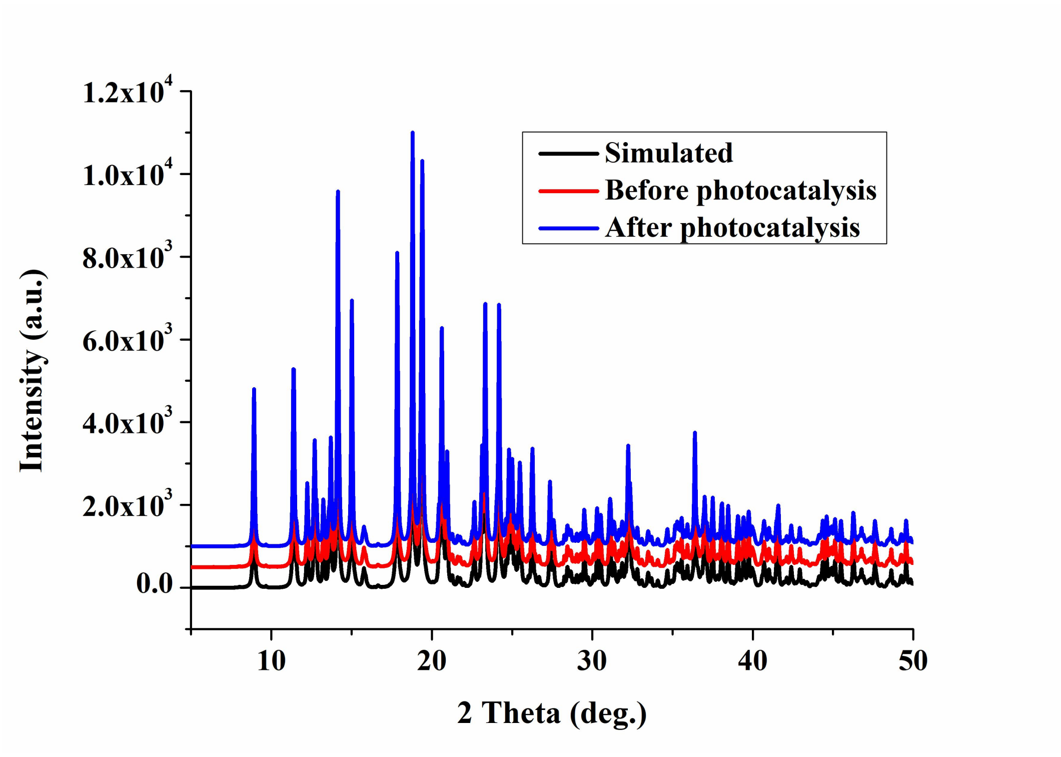

Figure S13. XRD of **1** before and after the experiment.

Table S1. Crystallographic Data of **1**.

	**1**
Empirical formula	$C_{18}H_{15}Cu_2N_6$
Formula weight	442.46
Temperature (K)	100
Wavelength (Å)	0.71073
Crystal system	monoclinic
Space group	$P2_1/c$
a (Å); *b* (Å); *c* (Å)	14.5080(7); 18.2789(6); 14.6503(7)
α (º); *β* (º); *γ* (º)	90; 107.953(5); 90
Volume ($Å^3$); *Z*	3695.9(3);16
D_{caled}, ($g.cm^{-3}$)	1.590
Limiting indices	$-14 \leq h \leq 18$; $-24 \leq k \leq 23$; $-20 \leq l \leq 18$
F(000)	1789
Θ range for data collection(º)	2.1 ~29.6
Reflections collected/unique	28575/9056
R_{int}	0.061
Crystalsize (mm^3)	0.11 ×0.10 ×0.08
$R[F^2 > 2\sigma(F^2)]$	0.048
$wR(F^2)$	0.162
S	0.89
$(\Delta/\sigma)_{max}$	＜0.001
$\Delta\rho_{max/}\Delta\rho_{min(}$e $Å^{-3}$)	1.02/-0.96

Table S2. Selected Bond Lengths (Å) and Bond Angles (°) for **1**.

**1**					
Bond	Dist. (Å)	Bond	Dist. (Å)	Bond	Dist. (Å)
Cu(1)-N(4)	2.044 (3)	Cu(2)-N(6)	2.192 (3)	Cu(3)-N(10)	2.048 (3)
Cu(1)-N(3)^{C}	2.158 (3)	Cu(2)-C(3)	1.961 (4)	Cu(3)-N(11)	2.049 (3)
Cu(1)-N(5)	2.054 (3)	Cu(2)-C(2)	1.951 (4)	Cu(3)-N(9)^{A}	2.009 (3)
Cu(1)-N(2)^{D}	1.987 (3)	Cu(2)-C(1)	1.964 (4)	Cu(3)-N(8)^{B}	2.121 (4)
Cu(1)-N(1)	1.969 (3)	Cu(4)-N(12)	2.186 (3)	Cu(3)-N(7)	1.975 (3)
Cu(4)-C(5)	1.947 (4)	Cu(4)-C(4)	1.969 (4)	Cu(4)-C(6)	1.966 (4)
N(1)-C(1)	1.140 (5)	N(2)-C(2)	1.158 (5)	N(3)-C(3)	1.159 (5)
Angle	(°)	Angle	(°)	Angle	(°)
N(11)-Cu(3)-N(10)	170.58 (13)	N(7)-Cu(3)-N(10)	89.00 (13)	N(5)-Cu(1)-N(3)^{C}	94.87 (13)
N(9)^{A}-Cu(3)-N(10)	88.32 (12)	N(7)-Cu(3)-N(11)	89.35 (12)	N(2)^{E}-Cu(1)-N(4)	90.46 (13)
N(9)^{A}-Cu(3)-N(11)	89.31 (12)	N(7)-Cu(3)-N(9)^{A}	155.26 (14)	N(2)^{D}-Cu(1)-N(3)^{C}	99.44 (13)
N(8)^{B}-Cu(3)-N(10)	93.77 (13)	N(7)-Cu(3)-N(8)^{B}	107.41 (14)	N(2)^{D}-Cu(1)-N(5)	87.04 (12)
N(8)^{B}-Cu(3)-N(11)	95.57 (13)	N(3)^{C}-Cu(1)-N(4)	96.27 (13)	N(1)-Cu(1)-N(4)	88.60 (12)
N(8)^{B}-Cu(3)-N(9)^{A}	97.31 (13)	N(5)-Cu(1)-N(4)	168.83 (13)	N(1)-Cu(1)-N(3)^{C}	105.77 (13)
N(1)-Cu(1)-N(5)	89.03 (13)	C(2)-Cu(2)-N(6)	98.11 (14)	C(1)-Cu(2)-C(3)	111.88 (16)
N(1)-Cu(1)-N(2)^{D}	154.73 (14)	C(2)-Cu(2)-C(3)	113.54 (15)	C(1)-Cu(2)-C(2)	120.47 (16)
C(3)-Cu(2)-N(6)	109.25 (14)	C(1)-Cu(2)-N(6)	101.18 (14)	C(5)-Cu(4)-N(12)	109.03 (14)
C(4)-Cu(4)-N(12)	97.66 (13)	C(6)-Cu(4)-N(12)	102.75 (14)	C(6)-Cu(4)-C(4)	106.63 (16)
C(4)-Cu(4)-C(5)	123.09 (16)	C(6)-Cu(4)-C(5)	114.58 (16)	C(3)-N(3)-Cu(1)^{F}	171.4 (3)

Symmetry codes: (A) $-x+2, y-1/2, -z+3/2$; (B) $-x+2, -y+1, -z+1$; (C) $x, -y+1/2, z-1/2$; (D) $-x+1, y-1/2, -z+3/2$; (E) $x, -y+1/2, z+1/2$; (F) $-x+2, y+1/2, -z+3/2$; (G) $-x+1, y+1/2, -z+3/2$.
